# Scaling Palaeolithic tar production processes exponentially increases behavioural complexity

**DOI:** 10.1038/s41598-023-41963-z

**Published:** 2023-09-07

**Authors:** Paul R. B. Kozowyk, Sebastian Fajardo, Geeske H. J. Langejans

**Affiliations:** 1 Department of Materials Science and Engineering, Delft University of Technology, 2628 CD Delft, The Netherlands; 2 Palaeo-Research Institute, University of Johannesburg, Johannesburg, Gauteng 2092 South Africa

**Keywords:** Archaeology, Archaeology, Social anthropology

## Abstract

Technological processes, reconstructed from the archaeological record, are used to study the evolution of behaviour and cognition of Neanderthals and early modern humans. In comparisons, technologies that are more complex infer more complex behaviour and cognition. The manufacture of birch bark tar adhesives is regarded as particularly telling and often features in debates about Neanderthal cognition. One method of tar production, the ‘condensation technique’, demonstrates a pathway for Neanderthals to have discovered birch bark tar. However, to improve on the relatively low yield, and to turn tar into a perennial innovation, this method likely needed to be scaled up. Yet, it is currently unknown how scaling Palaeolithic technological processes influences their complexity. We used Petri net models and the Extended Cyclomatic Metric to measure system complexity of birch tar production with a single and three concurrent condensation assemblies. Our results show that changing the number of concurrent tar production assemblies substantially increases the measured complexity. This has potential implications on the behavioural and cognitive capacities required by Neanderthals, such as an increase in cooperation or inhibition control.

## Introduction

Advanced cognition and behaviour in Pleistocene hominins are often studied based on the presence of what are considered complex technologies found in the archaeological record. It is generally assumed that the more complex a technology might be, the more modern the behaviour behind that technology is^1^. Thus, indicating when humans began to truly think and act as we do today. For example, heat treating of lithics, projectiles and weapons delivery systems, hafting, and trapping technologies are often seen as evidence for various stages in the development of complex cognition^2^. Such technical tasks rely heavily on expertise, a combination of working memory and long-term memory acquired over time, which may have been similar in both Neanderthals and modern humans^3, 4^. It has been suggested that a major difference between Neanderthals and modern humans potentially lies in the amount of innovation found among the two species^3, 5^. However, more evidence is slowly being brought to light demonstrating other technological developments or innovations among Neanderthals. These include the use of fire in the production of wooden spears and digging sticks^6^, fibre or cordage processing^7^, hafted hunting technologies^8^, and the production of different kinds of adhesives^9–11^.

Adhesives have received significant attention in discussions about Neanderthal complexity^9, 12–14^, and are agreed to have been a major innovation among both Neanderthals and modern humans^5, 15^. Adhesives contributed to other technological changes, such as hafting and composite stone tool production, facilitating the use of microliths, and later waterproofing vessels, containers, and boats^16^. It is also exceptional that the earliest known evidence of adhesives is associated with Neanderthals^17^, making it one of the few novel Neanderthal technologies that clearly pre-dates its use by anatomically modern humans. However, the circumstances that led to the discovery of tar, and the methods used by Neanderthals to make it when needed are still uncertain^9, 12, 13^.

Birch tar manufacture is unique among Palaeolithic technologies in that it requires the transformation of papery white bark into a black viscous and sticky liquid^18^, thus producing a novel material out of something entirely disparate for the first time. Much experimental work has gone into determining how birch bark tar could be produced using only materials and technology available during the Middle Palaeolithic^13, 19, 20^. Experiments indicate that Neanderthals could have discovered and re-discovered a number of tar production methods^19^. These include techniques such as the ‘condensation method’, ‘cobble groove’, ‘ash mound’, ‘pit roll’, and ‘raised structure’. Out of these known aceramic methods, the condensation technique is a likely candidate for one of the first way tar was discovered. It requires very few materials, little in the way of planning and preparation^21^, and produces strong tar^22^. The condensation process works by collecting airborne tar from the smoke of burned birch bark directly on stone surfaces^13^. Other production processes possibly used during the Palaeolithic to make tar operate differently. In the pit roll and raised structure methods, a small pit and vessel are used to limit oxygen and collect the tar product. The pit roll uses embers added directly on top of the birch bark to provide heat. The raised structure is more elaborate and uses a mesh of twigs to separate the bark from the cup in the pit below. An earthen dome is built around the bark to create an above ground oven-like structure and limit oxygen. A fire is built around the outside of the structure and is never in direct contact with the bark. These two methods differ from the condensation process in that they require an external fuel source and the tar drips into a collection vessel below the tar. One final technique, referred to as the cobble groove, requires multiple stones arranged in a chamber^23, 24^. Birch bark is placed inside the chamber and ignited. Tar condenses on the stones in the chamber, much as it does in the open-air condensation process, but because it is more closed, oxygen is further limited, more bark can be used at the same time, and tar yield is increased.

Methods such as the raised structure, pit roll, and cobble groove require more steps in the production process, as they have more individual components, which gives the impression that they are more complex. Based on the production steps, the condensation process appears to be the simplest^13^. It is possible that the condensation method and scaled up versions of this method gave rise to other the techniques. In a single condensation assembly, the tar yield per time and bark units is low. Thus raising concerns about how likely it was to have been used continuously and never improved on, especially when resources were scarce^9^. Experiments using three stones at once to condense tar speeds up the process and produces useable amounts of tar in shorter time-periods^25^. Further, the scaled-up condensation technique is a probable pre-cursor of the cobble groove method.

It is unknown what effect scaling the condensation process has on the complexity of the technological system, and how that impacts the amount of information that needs to be processed while making tar. Further, much of the current research lacks a systematic analysis of how changes between early tar production methods manifest into measured complexity^19, 24, 25^ (but see also^21^). To resolve this, we modelled tar production using Petri nets and measured changes in complexity associated with scaling the condensation method with a predefined metric. Petri nets are a model with formal definitions and are useful for analysing the information flow of systems^26^. Several metrics have been developed for modern applications to directly measure complexity and have been successfully used to measure the cognitive processes required for traditional adhesive making^21, 27^. Here we use the Extended Cyclomatic Metric (ECyM) to measure and compare the complexity of a single condensation process (using one cobble; CONx1), with that of three concurrent production assemblies (using three cobbles at the same time; CONx3). A higher ECyM score indicates a more complex process with more decisions and greater reliance on inhibitory control, a higher order executive function^21^. The Petri net models also show the amount of information embedded in each technological system^27^. Combined, our study sheds light on what cognitive or behavioural traits were likely required by Neanderthals to develop tar starting from the condensation method.

## Methods

### Experimental tar production

There are many possible ways to produce tar from biomass. Today, tar is still made from wood and bark using metal or ceramic containers and an external heat source^28, 29^. At raised temperatures and in a reducing environment, organic material undergoes thermal decomposition and forms tar, charcoal, and other non-condensable gases. This process can also be carried out using only Palaeolithic technology without ceramics or metal retorts by creating an earthen mound or ‘raised structure’ above a small pit in the ground^19^. However, a distinct method of producing tar from birch bark is also possible without relying on a reducing environment. Referred to as the ‘condensation method’, this technique uses no containers, pits, structures, or external fuel sources. The method requires a condensation assembly that is composed of small pieces of birch bark burned next to a near vertical smooth stone surface, such as a river cobble. As the bark burns, a black tarry substance condenses on the stone surface and can be intermittently scraped off using a flint flake^13^.

We reproduced the condensation process and created a Petri net based on observations and recordings. During our experiments, the following steps were identified, which together make up the process from start to finish, when the first piece of tar is stored. The cobble used to condense the tar is placed in a suitable location, with one flat face at an angle ⪆ 90° to the ground. Bark is torn into small pieces a couple of cm wide and approximately 10 cm long. The bark is lit on fire from an external fire source and placed at the base of the cobble. From this point, three things can happen: the bark extinguishes prematurely, in which case it is reignited and placed again at the base of the cobble; the bark falls down or moves, in which case it is repositioned again closer to the base of the cobble; or the bark burns successfully and tar begins to condense on the cobble surface. After this happens, the bark was either held in place closer to the rock to facilitate more condensation or left on its own to burn. Once enough tar condenses on the rock, or when the bark burns up, tar is scraped from the surface of the cobble using a flint flake. Finally, the tar is collected off the flint flake and stored to the side. The process is then repeated in the same way until enough tar is collected to use.

We used a single condensation method to model the production process^21^, and then extended the model to recreate a production event that scaled up the condensation process in a single event using three assemblies from^25^. This allows a direct comparison of the smallest possible condensation assembly (comprised of a single cobble) with a realistically up-scaled assembly (comprised of three cobbles). We used three assemblies because it has been documented in the literature and is reported as successfully increasing the yield/time of the condensation method. More than three cobbles was not always manageable by a single experimenter, depending on the quality of bark being used^25^. Further, neuroscientific experiments have shown that the reliance on working memory capacity in natural modern human behaviour limits tasks to less than four items^30^. Three cobbles therefore falls within this range, and is a realistically scaled up condensation process for Neanderthals to have been capable of using.

### Modelling

The Extended Cyclomatic Metric (ECyM), developed from the Cyclomatic Metric (CM) originally designed to test for the likelihood of errors in complicated pieces of code^31^, is associated with the underlying behavioural complexity of the tar production process. More choices in a production process create more possible paths to the end goal, which increases the likelihood of errors, resulting in deadlocks, which result in wasted time and resources^21^. We use the ECyM because we are interested in how the complexity of the behaviour of the system changes as a result of scaling the process by adding more cobbles. A higher ECyM score indicates that there are more possible paths to process completion, which increases the possibility for errors. Inhibitory control helps to avoid the repetition of errors, so ECyM can be used as a proxy for the amount of inhibitory control required^21^.

**Figure 1:**
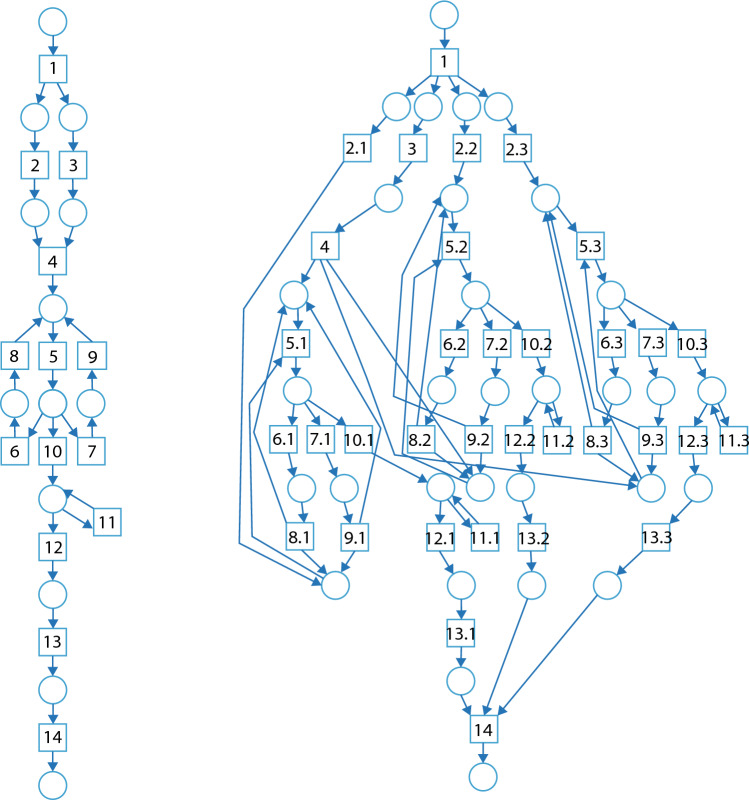
Workflow nets of a single condensation assembly (CONx1; left) and three concurrent condensation assemblies (CONx3; right). Transitions, represented by squares, correlate to actions or events that change the state of the system. Identified during experimentation, these are: (1) start, (2–2.3) place rocks, (3) tear bark, (4) light bark, (5–5.3) place lit bark, (6–6.3) bark moves, (7–7.3) bark extinguishes, (8–8.3) grab bark, (9–9.3) reignite bark, (10–10.3) start condensation, (11–11.3) hold bark, (12–12.3) stop condensation, (13–13.3) scrape tar, (14) store tar.

In order to create the models for the ECyM, two distinct production events, one with a single tar condensation assembly (CONx1) and one event consisting of three production assemblies (CONx3) were modelled as Petri nets using Snoopy 2 version 1.22^32^ (Fig. 1). Petri nets are a formal modelling language with underlying mathematical semantics that are used in process modelling to study system complexity^26, 33^. A petri net contains three basic elements: places, transitions, and arcs that are graphically represented as circles, rectangles, and arrows respectively. Places represent conditions, locations or buffers between events, transitions represent events that change the state of the system, such lighting birch bark on fire, and arcs show causal relationships and the flow of the system^21, 34^. As the premise and materials are the same between the single and triple condensation processes, we assume that all tools, and resources including fire are already obtained, and that social, cultural, environmental, and time restrictions do not affect the workings of the production process itself. Each Petri net models the process until the first tar is collected and stored. After this point, each process would likely need to be repeated until enough tar is collected to use^21^. However, this does not affect the complexity as the cognitive load is reset once the process is started again.

**Figure 2:**
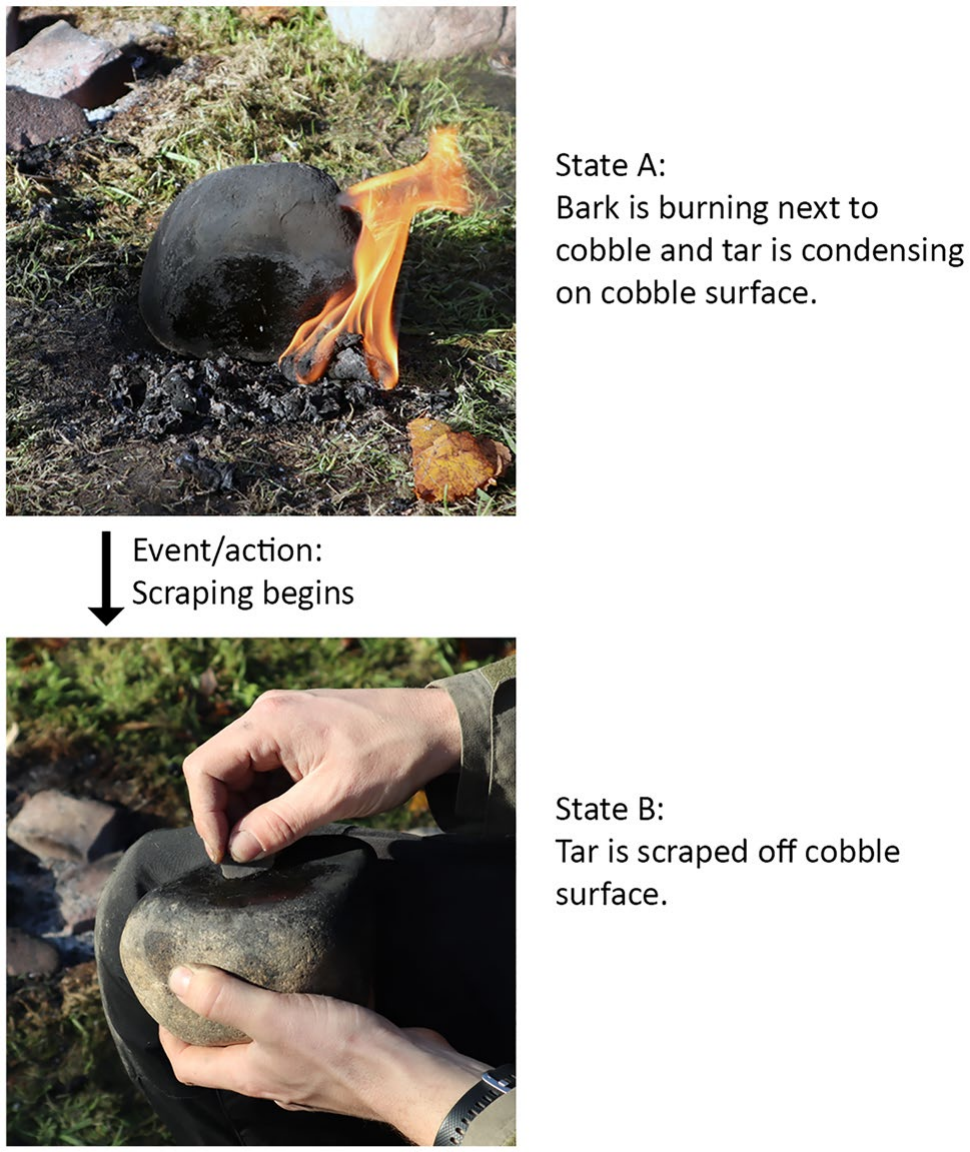
Event altering the state spaces of the system. A state is a unique instance in time, represented here by each photograph. Events or actions that can change the state from one form to another are represented by the arrow between each photo. The state space represents all possible states between the beginning and end of the entire process.

Petri net models can identify the number of different states that a modelled system can reach, which serves as a proxy for the amount of information embedded in that particular system^21, 27^. A state is a unique instant in time, or a snapshot, in which we can observe the system. In a Petri net, the causal relations between transitions and places form specific conditions within different parts of the process at different moments in the workflow. A set of fulfilled conditions at a given moment of the process defines a reachable state. All available, or reachable states form the state space of the system. The relationship between resources and actions define how states change from one to another, and determines the number of states and paths from the initial to the final state of the system. For example, in the following ‘snapshot’, birch bark is burning next to a cobble (Fig. 2). Beginning to scrape tar from the cobble surface changes the state from one form to another. Work-flow nets, a sub-class of Petri nets are used to model these processes^21, 31^, to allow a formal comparison of the sizes of the state space between different production systems.

The larger a state space is, the greater the number of configurations that the system can reach. This in turn shows that the potential information embedded in the structure of the production system is also greater. More information will therefore need to be processed to get from one state to the next in order to reach the final state and end the process. On the other hand, production systems with smaller state spaces will require processing less information before reaching the final state. When the state space of a Petri net model has a finite number of reachable states, as is the case in this study, the state space can be expressed as a reachability graph to count the states of the system. This provides quantitative data on the complexity of the process. Reachability graphs depict the sets of conditions that become satisfied following the occurrence of an event. An event can activate one or multiple conditions, leading to a new reachable state. It can also maintain the system in its existing state, which is the case with self-loop events. Reachability graphs represent these relations, facilitating the identification of how events enable specific states to reach subsequent states. A large state space in the reachability graph represents a process with a high complexity because there are more possible paths to reach the end of the process^27^.

**Figure 3:**
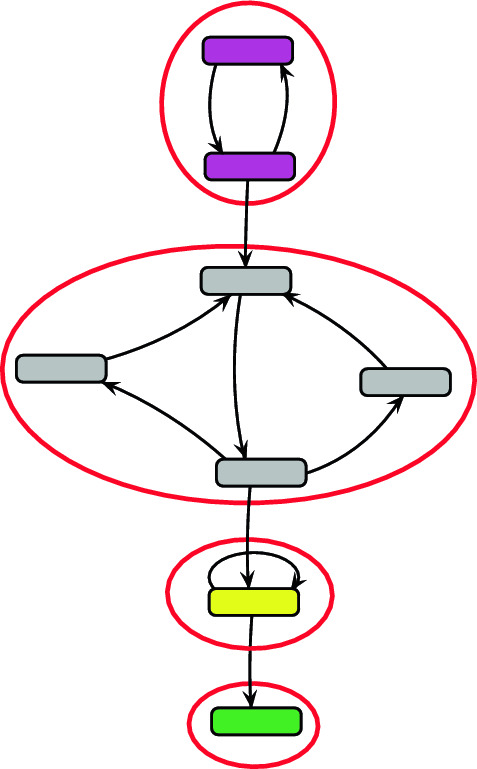
Strongly connected components in an arbitrary reachability graph. Each red circle encompasses what is considered as a SCC. Within each SCC there is a pathway from each vertex to another vertex. In this case the largest SCC is shown in grey, and the smallest in green.

Cycles also affect the complexity of a system and the desired properties of its products. For example, traditional production of *A. coranica* adhesives shows cycles of pounding and kneading to obtain an adhesive product out of a single component^35^. Likewise, the number of sinew coating cycles of bows done by Assiniboine makers determine how strong the bow will be^36^. In a reachability graph, these types of cycles are identified as strongly connected components (SCC). SCCs are formed by a set of reachable states when, for every pair of states x and y, there is a path leading from x to y^31^. The minimal SCC is represented by a single reachable state. Figure 3 shows examples of SCCs in an arbitrary reachability graph. Within the largest SCC shown in grey (Fig. 3), there is a possible path from any one of the transitions to the others. There is no possible path back to the pink SCC, or from the yellow back to the grey, so they are separate. The ECyM takes into account both the complexity represented by the number of reachable states and the number of SCCs during the processing of the system in a single calculation, making it the ideal metric to be used in this situation. The ECyM is calculated by the equation^31^:

$$ECyM = \, \left| E \right| \, - \, \left| V \right| \, + p$$

where* E* is the total number of events (edges), *V* is the total number of reachable states, and *p* is the number of SCCs in the reachability graph. For further information on Petri nets and the ECyM, see ^31^.

## Results

**Figure 4:**
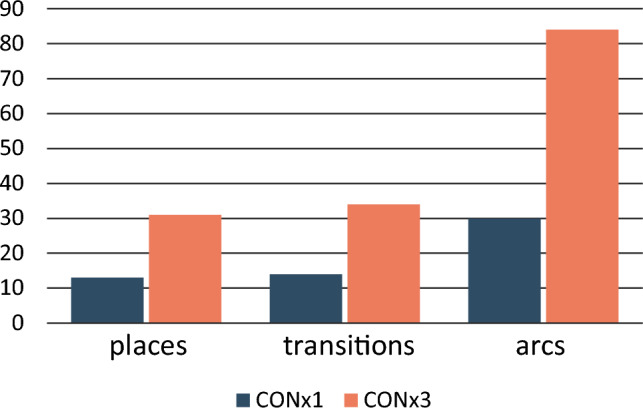
Number of places, transitions, and arcs of the condensation methods with one cobble (CONx1; blue) and three cobbles (CONx3; orange). The number of places and transitions increase in similar proportions (by a factor of 2.5 and 2.4 respectively), and the number of arcs increases by a higher factor of 2.8.

The CONx1 model contains 12 places, 14 transitions, and 30 arcs while the CONx3 model contains 31 places, 34 transitions and 84 arcs (Fig. 4). The ratio between places and transitions occurs in similar proportions in the Petri nets of both production methods. However, the CONx3 contains proportionately more arcs relative to the places and transitions. This is expected because while the number of cobbles increased from one to three, the transitions ‘Start’, ‘Light bark’, and ‘Store tar’ and adjacent places are all shared by branches created by the three cobbles. Therefore, the number of places and transitions should all increase by less than a factor of three, and the number of arcs should increase more than the number of places and transitions.

**Figure 5:**
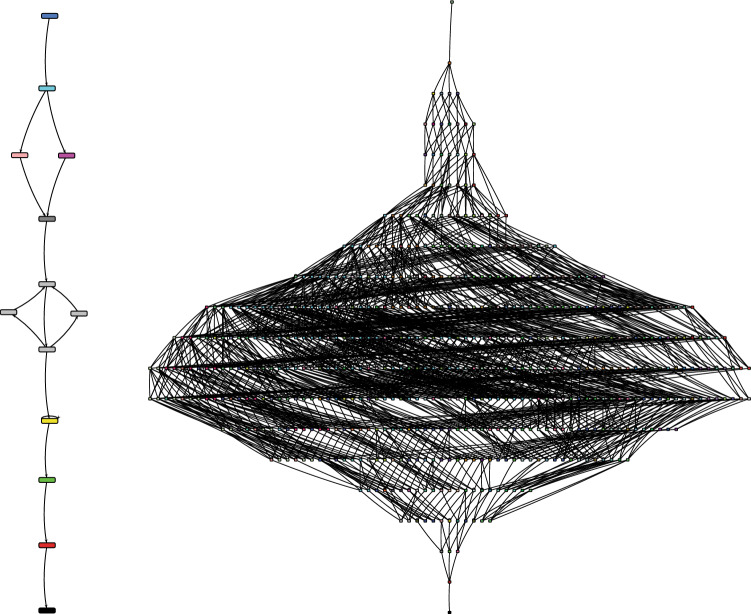
Reachability graphs for CONx1 (left) and CONx3 (right). Graphs show all possible paths (edges, E) that can be taken to process completion and the total reachable states (V). Each unique SCC (p) is represented by a different colour. For CONx1 E = 16, V = 13, and p = 10, giving an ECyM score of 13. For CONx3, E = 1962, V = 530, and p = 143, giving an ECyM score of 1575.

CONx1 has 16 edges, 13 reachable states, and 10 SCCs, and CONx3 has 1962 edges, 530 reachable states and 143 SCCs (Fig. 5). The ECyM scores calculated from the reachability graphs is 13 for CONx1 and 1575 for CONx3; an increase of over two orders of magnitude.

## Discussion

Our results show that while the number of places, transitions, and arcs all increase by a ratio of less than 1:1 with the number of production assemblies, the ECyM describes a much larger increase in complexity. By only looking at changes in the number of steps in the process, or the materials required, archaeologists could mistakenly conclude that scalability has little additional impact on complexity. An increase from one to three concurrent assemblies results in the number of transitions, or steps in the process, increasing from 14 to 34. However, because the ECyM also considers the number of reachable states and SCCs it shows how these changes in the process manifest into the complexity of the structure of the system as a whole. The increase in ECyM values from 13 to 1575 clearly indicates that we cannot simply suggest upscaling a technological process to increase efficiency (cf.^25^) without also considering how that will influence the entire system.

To put the condensation ECyM scores in perspective, the highest values of the ECyM calculated from 262 selected modern processes was approximately 7000 and the minimum value was approximately 5, with a mean of 169.94^31^. The raised structure tar production method, which is the often regarded as the most ‘complex’ of the known Palaeolithic methods^19^, has an ECyM score of 38^21^. While CONx1 has ECyM = 13 and CONx3 ECyM = 1575. When compared with the sample of modern processes, most tar production methods have a relatively low ECyM score. However, scaling the condensation up to three concurrent assemblies increases the score to well above the mean for modern processes.

Such an increase in ECyM complexity is indicative of the number of paths that can be taken, which require decisions to be made during the process. These decisions, in turn, have implications for how successful the production process is. There is a higher likelihood of problems arising when a single individual is responsible for processing all the information of three cobbles at the same time. For example, if tar is scraped and stored while another roll of bark extinguishes, then that bark will need to be reignited. If tar is left too long on the surface of one stone, it may burn away, reducing the yield efficiency of the process. A higher number of possible paths, or choices that need to be made during the process, means that there is a higher risk of failure, wasting both time and resources^21^. Inhibitory control is required to ensure that errors in the process are not repeated^21^. Further, the increase in reachable states from 13 (CONx1) to 143 (CONx3) indicates that much more information is embedded in the system of the latter^27^. Because time is constrained primarily by the speed at which the bark burns, when a single individual is responsible for processing the extra information in CONx3, they use their attention capacity intensely on the task at hand. Both inhibition and attention are strongly linked with cerebellar volumes in the brain^4^, which may have been smaller in Neanderthals than modern humans^37^ (but see also^4^).

Neanderthals may have solved the problem of coping with processes requiring higher attention and inhibition control by dividing the task among multiple individuals and working together. For example, individuals may establish social systems to distribute cognitive loads of complex tasks that exceed their cognitive capacities^38^. It has also been suggested that costs associated with collecting fuel and maintaining fire required cooperation among individuals within groups^39^. Further, the spread of the controlled use of fire around the middle of the Pleistocene may also indicate modern-like human social interactions between groups^40^ (but see also^41^). Dividing the cognitive load of concurrent tar production assemblies among multiple individuals would also have practical benefits concerning fire use. It would limit the possibility of a fire going out while attention is focused by a single individual on specific tasks within the condensation process, such as scraping and storing the tar. In our experiments, the fire extinguished and needed to be reignited from an external source 16 times during a 76-min production sequence. Fire from an external source is also introduced twice during a 6 min video of a single condensation tar production^13^, and two times in a 30 min video, with a lighter being used a total of 16 times to ignite bark during the same period^25^. Under optimal warm and dry conditions with high quality prepared tinder and kindling, a flame can be produced using pyrite and flint in as little as one minute. Under less ideal conditions it may take up to five minutes (personal communication, Sorensen, A., 16-11-2021). However, the tinder material itself is also valuable and constitutes another level of investment to prepare and store. A separate fire may be used to provide a flame source for re-igniting the birch bark as required, and this is the simplest solution, but then the mental and material resources used in maintaining such a fire need to be considered as well. Having two or more individuals producing tar concurrently with the condensation process greatly reduces the chances of having all of the birch bark extinguish at the same time.

It is also possible that instead of scaling tar production processes, Neanderthals developed novel production methods, such as the pit roll or raised structure, which have lower ECyM values than CONx3. Birch tar possibly being produced in a pit roll or raised structure at Königsaue, Germany^42^, would support this. Our results clearly show how increasing concurrent production assemblies can greatly influence complexity. This ultimately has implications for the cognitive and/or behavioural requirements of Neanderthals. If inhibition and attention were limited in Neanderthals, they may have relied on alternatives such as social cooperation to solve problems, or innovated and devised new ways of producing tar to overcome the limitations the condensation technique posed to them.

## Conclusion

Petri net models are a useful tool for measuring and comparing the complexity of ancient adhesive production processes^21, 27^. Here we have shown that the ECyM is valuable for measuring the effects of scalability on process complexity and underlying behaviour. Our results comparing ECyM scores of single and concurrent assemblies have two important outcomes: (1) small changes in the process, such as increasing the number of concurrent production assemblies from one to three, significantly increases the measured complexity. Care must therefore be taken when describing the complexity or behaviour of such processes without considering the full impact of up-scaling. (2) The extremely high ECyM score, and the high number of reachable states of three concurrent condensation production assemblies suggests that if this method were employed, some form of behavioural adaptation, such as social cooperation, may have been employed to mediate the inherent problems associated with its high complexity. Finally, this method of analysis has potential implications beyond comparing tar production techniques. Other technological processes that contain concurrency may also have had high ECyM complexity and required similar solutions to overcome.

## Data Availability

All relevant data is available within the manuscript.
